# Design and Elementary Evaluation of a Highly-Automated Fluorescence-Based Instrument System for On-Site Detection of Food-Borne Pathogens

**DOI:** 10.3390/s17030442

**Published:** 2017-02-23

**Authors:** Zhan Lu, Jianyi Zhang, Lizhou Xu, Yanbin Li, Siyu Chen, Zunzhong Ye, Jianping Wang

**Affiliations:** 1 College of Biosystems Engineering and Food Science, Zhejiang University, Hangzhou 310058, China; 11413014@zju.edu.cn (Z.L.); zhangjianyilong2@126.com (J.Z.); xu.lizhou@163.com (L.X.); sychen412@gmail.com (S.C.); zzye@zju.edu.cn (Z.Y.); 2 Department of Biological and Agricultural Engineering, University of Arkansas, Fayetteville, AR 72701, USA; yanbinli@zju.edu.cn

**Keywords:** food-borne pathogens, fluorescence, automated instrument system, on-site detection, quantum dots, LabVIEW

## Abstract

A simple, highly-automated instrument system used for on-site detection of foodborne pathogens based on fluorescence was designed, fabricated, and preliminarily tested in this paper. A corresponding method has been proved effective in our previous studies. This system utilizes a light-emitting diode (LED) to excite fluorescent labels and a spectrometer to record the fluorescence signal from samples. A rotation stage for positioning and switching samples was innovatively designed for high-throughput detection, ten at most in one single run. We also developed software based on LabVIEW for data receiving, processing, and the control of the whole system. In the test of using a pure quantum dot (QD) solution as a standard sample, detection results from this home-made system were highly-relevant with that from a well-commercialized product and even slightly better reproducibility was found. And in the test of three typical kinds of food-borne pathogens, fluorescence signals recorded by this system are highly proportional to the variation of the sample concentration, with a satisfied limit of detection (LOD) (nearly 10^2^–10^3^ CFU·mL^−1^ in food samples). Additionally, this instrument system is low-cost and easy-to-use, showing a promising potential for on-site rapid detection of food-borne pathogens.

## 1. Introduction

Food safety is one of the major health concerns that severely threatens human health and could lead to tremendous loss in society. Food-borne infections in the United States alone have been estimated to cause ~76 million illnesses, more than 300,000 hospitalizations and 5000 deaths each year [1]. Moreover, food contamination could result in a loss of 25% of food produced every year [2]. Bacterial pathogens, such as *Escherichia coli* O157:H7, *Salmonella*, *Campylobacter jejuni*, and *Listeria monocytogenes*, are the leading causes of these bacterial foodborne illnesses [3]. Recently we have witnessed continuous outbreaks of food social events attributed to foodborne pathogens, such as the enterohaemorrhagic *E. coli* (EHEC) contamination of sprouts in Germany in 2011, a *Listeria monocytogenes* outbreak in cantaloupe melons in the United States in 2012 [4], and *Salmonella* infection in Europe in 2012 where 65,317 cases were reported by the European Centre for Disease Prevention and Control (ECDC) [5]. In order to reduce the occurrence, there is an ever-increasing urgency of more rapid and easy-to-use methods for pathogen detection.

Conventionally, culture-based methods are the basic tools used for detection of food-borne pathogens around the world for their reliability in efficiency, sensitivity to target organisms, and application to a wide range of food matrices (e.g., all food and feedstuffs) and environments of food production and handling [6]. Yet it is a time-consuming process [7] and needs trained operators with a considerable amount of laboratory equipment. Alternative rapid, but accurate, methods therefore have been sought to overcome these limitations [8] and the existing literature has reported numerous methods. Generally, these rapid methods are categorized into nucleic acid-based, immunological-based, and biosensor-based methods [9]. Some representative methods, such as polymerase chain reaction (PCR) [10,11], enzyme-linked immunosorbent assay (ELISA) [12], surface plasmon resonance (SPR) [13], and impedimetric biosensors [14], have already been able to detect various kinds of food-borne pathogens in food samples with close accuracy in a shorter time, yet each has its own advantages and limitations. Among them, considerable attention has been paid to the methods based on fluorescence using semiconductor nanocrystal quantum dots (QDs) as fluorophores coupled with magnetic nanoparticles for separation. Magnetic nanoparticles could efficiently separate samples from the liquid suspension. Magnetic separation has been widely used as a universal separation tool to purify nucleic acids (i.e., DNA and RNA), proteins and peptides, cells, and other biologically-active compounds from crude samples [15]. The fluorescence-based method is well-known for its high sensitivity, especially using QDs as fluorophores, which could offer high brightness, long fluorescence lifetime, and good photo-stability [16], compared to traditional fluorescent labels. A number of remarkable studies in this method have been done [17,18,19]. The latest result, to our knowledge, showed simultaneous detection of four food-borne pathogens (*E. coli* O157:H7, *S. aureus*, *L. monocytogenes*, and *S. Typhimurium*) with a limit of detection down to 80, 100, 47, and 160 CFU·mL^−1^, respectively, in pure culture, and 320, 350, 110, and 750 CFU·mL^−1^ respectively in ground beef, which shows great potential to meet the basic requirements for in-field application [20].

To step further to convert this novel and effective method into practical use [20,21], attention should be paid to the development of portable, highly-automated, and simple-to-use instruments. Yet, at present, the majority of papers reported are concerned with experimental method in lab use [19,20,21,22,23,24,25,26], such as taking advantage of novel protocols to achieve simultaneous detection for more types of pathogens [22], exploring more types of QDs with different emission wavelengths [25], or designing new types of conjugation systems to amplify fluorescence intensity [26]. In their work, spectral signal acquisition was generally done by ready-made laboratory instruments, with a hand-made cuvette holder in a dark box [20], or other likely methods for sample positioning. The whole procedure, from sample switching to spectrum collecting and data processing were all operated by hand. It could not satisfy the utilization in a practical environment where high-throughput and high-uniformity for detection conditions are critically required. Additionally, the instrument used on the spot should also be compact, easy-to-use, and show stable performance.

In this study, we have developed an integrated instrument system, including apparatus hardware and analysis software, coordinated with developed biosensing methods for rapid, portable and highly-automated application in food safety. Herein we present the design and fabrication of every module of this instrument system in detail. We demonstrated this system’s capabilities through a series of evaluations, especially by the comparison with a widely used commercial product. Elementary bacteria tests were also be included for validation. Furthermore, the availability of this home-made system for on-situ use was discussed at last. To our knowledge, this paper is the first study in developing a portable instrument in the point of instrument based on fluorescence using QDs as fluorophores for multiplex food-borne pathogen on-site detection.

## 2. Materials and Methods

### 2.1. Quantum Dot Sample Preparation

Three CdSe/ZnS core/shell QDs with emission wavelengths of 528, 572, and 621 nm were purchased from Ocean NanoTech (San Diego, CA, USA). The concentrations of the three QDs were all 1 μM.

Phosphate buffered saline (PBS, 0.01 M, pH 7.4) were purchased from Sigma-Aldrich (St. Louis, MO, USA). It was autoclaved at 121 °C for 30 min before use.

For the pure QDs test, the original QD solution was diluted using PBS solution to desired concentrations. Then the QD samples (1, 1 × 10^−1^, 1 × 10^−2^, 1 × 10^−3^, 1 × 10^−4^ μM) were put in the transparent glass tubes, respectively, ready for fluorescence measurement in the system evaluation.

### 2.2. Bacteria Sample Preparation

Three typical kinds of food-borne pathogens, *E. coli* O157:H7 (ATCC 43888), *L. monocytogenes* (ATCC 43251), and *S. Typhimurium* (ATCC 14028), were purchased from the American Type Culture Collection (ATTC, Manassas, VA, USA). To simulate a practical environment, we used lettuce bought freshly in local grocery stores prior to tests to be inoculated with the bacterial pathogens. Complete preparation steps, including pretreatment, inoculation, immunomagnetic separation and QD conjugation had been reported in our previous paper [20]. Herein, levels of target pathogens were 10^0^–10^7^ CFU·mL^−1^ and PBS was used as the negative control in the evaluation of this home-made instrument in the aspect of pathogens detection.

### 2.3. Apparatus and Software

Fluorescence measurement and data analysis were conducted using this developed instrument system. For comparison, the fluorescence intensity of pure QDs sample was detected in parallel by a commercial product, Synergy H1 Multi-Mode Microplate Reader with Gen5 2.0 Data Analysis Software (BioTek Instruments, Winooski, VT, USA). The Synergy™ H1 is a flexible monochromator-based multi-mode microplate reader that can be turned into a high-performance hybrid system with the addition of a filter-based optical module. This instrument has been widely used as a credible protocol for fluorescence analysis in relevant research [27,28,29].

## 3. Instrument System Description

**Figure 1:**
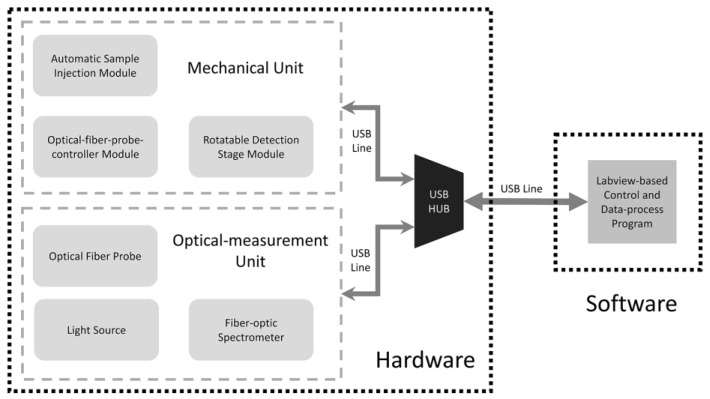
The structure of the whole instrument system.

This instrument system was composed of two typical parts, hardware and software, as illustrated in Figure 1. 

The hardware consisted of two main units, a mechanical unit and an optical measurement unit. The mechanical unit mainly included three stepper motors with their controlling components, a rotation stage in which tubes containing samples were placed and other additional controlling accessories. This unit served to drive the whole system to run automatically in each detection run. The optical measurement unit consisted of several commercial off-the-shelf components, including the light source, optical fiber probe, and fiber-optic spectrometer. The optical fiber probe transferred the light from the light source to the sample, and then collected the fluorescence signal from the sample excited by the light source to the spectrometer. The software was developed based on LabVIEW (v2012, National Instruments, Austin, TX, USA), and was served as an interface for the operators to control the mechanical unit to do each specific action. In the meantime, it could collect spectral data from the optical measurement unit, giving out the final detection results after a series of data handling processes. Hardware and software were connected with a USB communication line via the USB port on the computer.

The hardware was covered by an aluminum extrusion-made shell to protect it from the environmental visible light interference, except for a small hole on the rear near the bottom of the backside of the unit for the USB communication cable to pass through.

In this system, two units in the hardware should be connected to the software separately. Thus, a USB hub was used so that only one USB port on the computer would be populated.

### 3.1. Hardware

#### 3.1.1. Optical Measurement Unit

**Figure 2:**
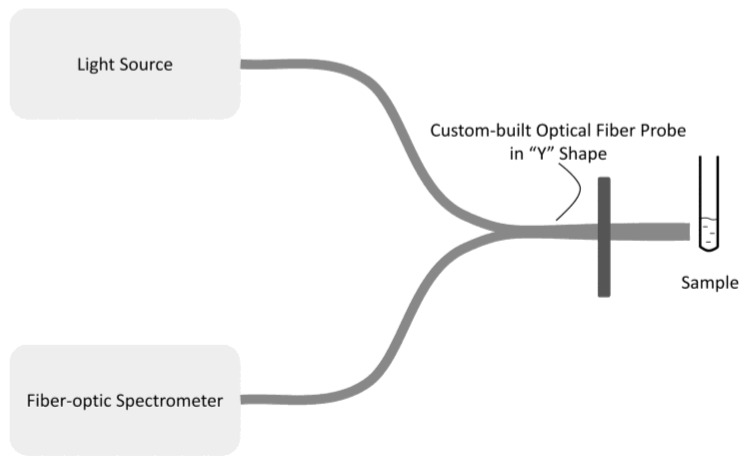
The structure of optical measurement unit.

The structure of this unit is described in Figure 2. The three major components comprising this unit were the light source, the optical fiber probe, and the fiber-optic spectrometer.

The highly-sensitive LED supplied light with a central wavelength at nearly 450 nm (model LS-450, Ocean Optics, FL, USA) to the disposable borosilicate glass tube (part number 73500-650, Kimble Chase, TN, USA), in which there were to-be-detected samples conjugated with QDs, through an optical fiber probe (SPL Photonics, Hangzhou, China). The fluorescence excited from the QDs in the sample would be collected through the other branch of the optical fiber probe to the fiber optic spectrometer (model USB4000, Ocean Optics, Dunedin, FL, USA), which was powered by the USB port on the computer. Then this analog spectral data would be digitized by the fiber optic spectrometer and, finally, the complete fluorescent spectra of this sample would be transferred to the software also via the USB port. 

**Figure 3:**
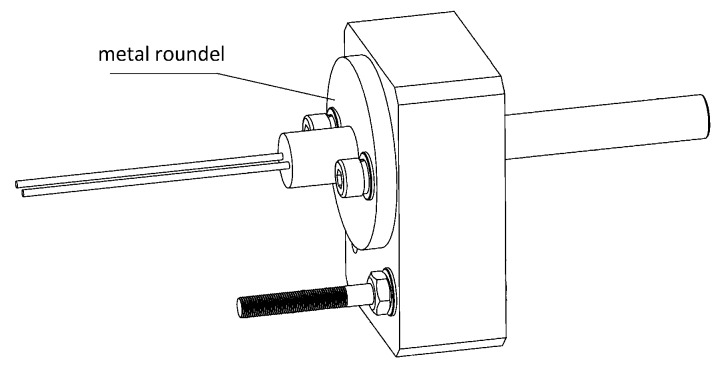
Detailed configuration of this custom-built optical fiber.

The optical fiber probe was custom-built in a “Y” shape, as shown in Figure 2, two branches divided near the light source, one directly pointing to the sample and the other connecting with the fiber optic spectrometer. A metal roundel with two thread holes was customized to be fixed to the branch of the optical fiber probe pointing to the tube, so the fiber probe could be fixed with the optical-fiber-probe-fixed block by two hex bolts, as depicted in Figure 3, and now the position of the optical fiber could be controlled by the mechanical unit.

The spectrometer USB4000 we used in this system was responsive from 200 to 1100 nm, which covers the excitation wavelengths that are nearly 528 nm, 572 nm, and 621 nm of the QDs we used. This kind of spectrometer had been proven to be able to collect the fluorescence signal excited from QDs in our previous research [1]. 

#### 3.1.2. Mechanical Unit

**Figure 4:**
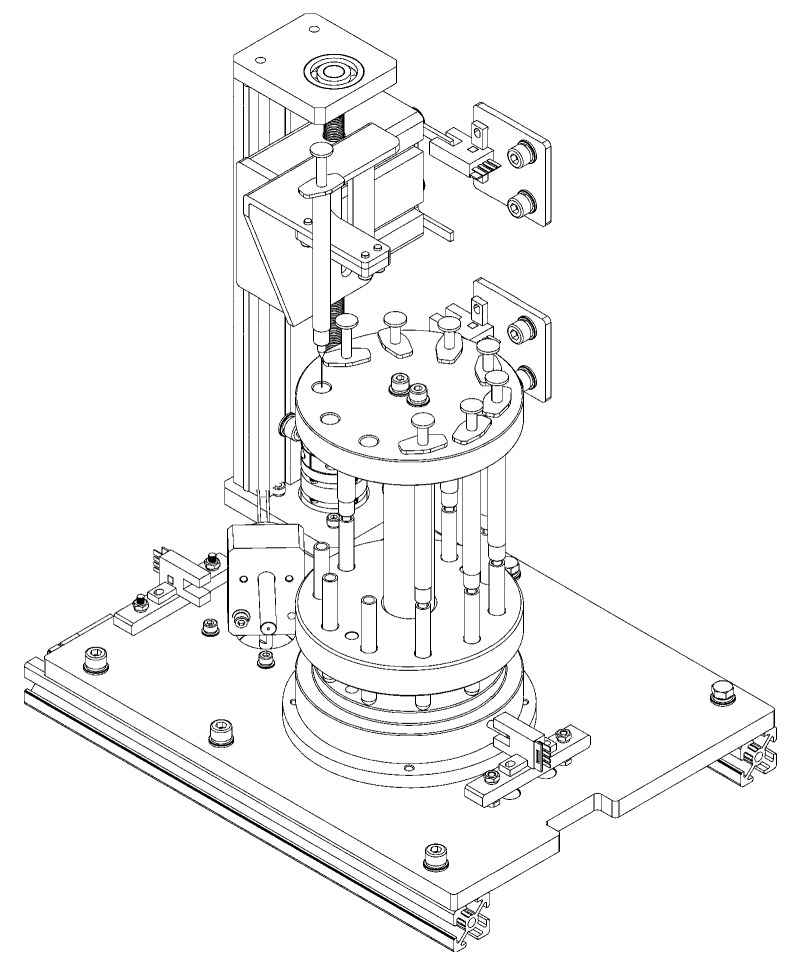
Appearance of the mechanical unit in a 3D sketch.

Figure 4 shows an appearance illustration of this unit in 3D, which was comprised of the following three parts in total: a rotatable detection stage module, an automatic sample injection module, and an optical fiber probe controller module. The detection stage module was placed in the bottom. The injectors and the tubes containing samples to be detected were put inside, and the rotatable sample-positioned stage herein could revolve around the center to switch each sample under detection one by one. The automatic sample injection module was used to press the samples in the injectors into the tubes right beneath them by the up and down movement of the injector compression plate. The optical fiber probe controller module was to control the station of the optical fiber probe as mentioned in Section 3.1.1.

In this unit, an Atmega 16A (Atmel, San Jose, CA, USA) was placed on the back. This is a high-performance, low-power AVR eight-bit microcontroller composed of 16 KBytes of ROM, 1 KB SRAM, and four pulse width modulation (PWM) channels. It functioned as the brain in this device. Three stepper motors (Leadshine, Shenzhen, China), one in each module, together with their respective actuators, were directly connected to the microcontroller via the IO port. Each stepper motor actuator could output a different PWM signal to drive its respective motor to run according to the command from the microcontroller. With the cooperation of other accessories, such as the coupling with a leadscrew, photo-electric switch sensors and so on, these three modules were able to automatically accomplish their own assignments, and then the whole unit could run without extra manual operation in each single detection run.

The communication between this unit and computer was also in charge of the microcontroller, using the UART (universal asynchronous receiver/transmitter) in it.

##### Rotatable Detection Stage Module

**Figure 5:**
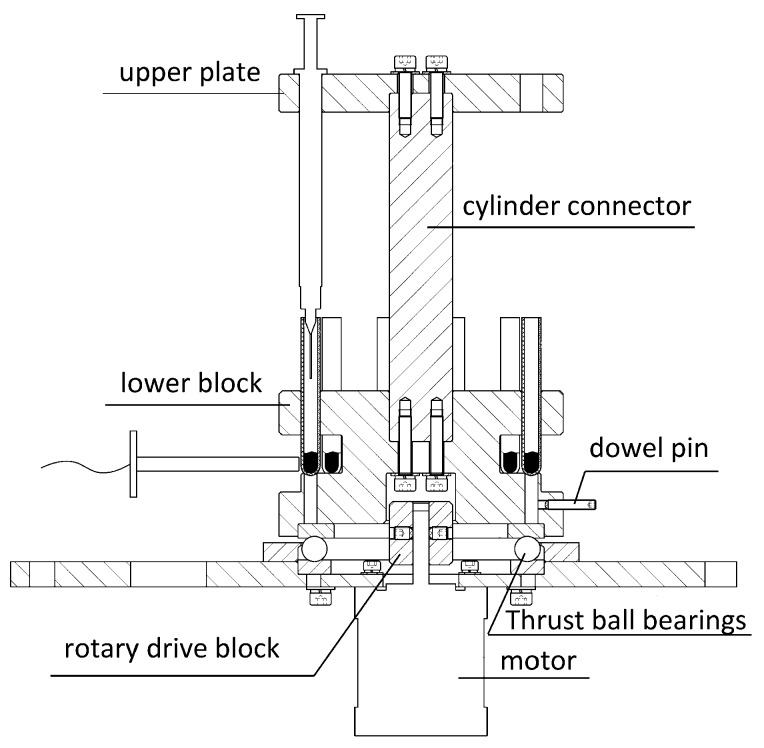
Side section drawing of the rotatable detection stage module.

Figure 5 shows the side section drawing of the rotatable detection stage module.

The rotatable sample-positioned stage made of aluminum was a principal component in this module. This stage was assembled with three elements: the upper plate with holes evenly distributed along the circumference to position one-off injectors, the lower block with holes distributed in the same way immediately beneath to position tubes, and the cylinder connector placed between them to connect them with hex bolts. The whole stage was placed on the base of the instrument, embedded into a rotary drive block fixed on the shaft of the driver motor by a rectangle-shaped hole on the bottom of the lower block. Thus, the stage could be driven to rotate together with the rotary drive block by the rotation of the motor. At the same time, it was able to be taken out to exchange injectors and tubes. Thrust ball bearings were placed between the stage and the base, making it smoother in rotation. A dowel pin was mounted on the under lateral of the lower block, together with a photo-electric switch sensor installed on the base of the instrument near it for orientation. In the spinning of the stage, the instant the dowel pin triggered the photo-electric switch sensor, the microcontroller would stop the motor and the stage now reached the initial working position.

##### Automatic Sample Injection Module

**Figure 6:**
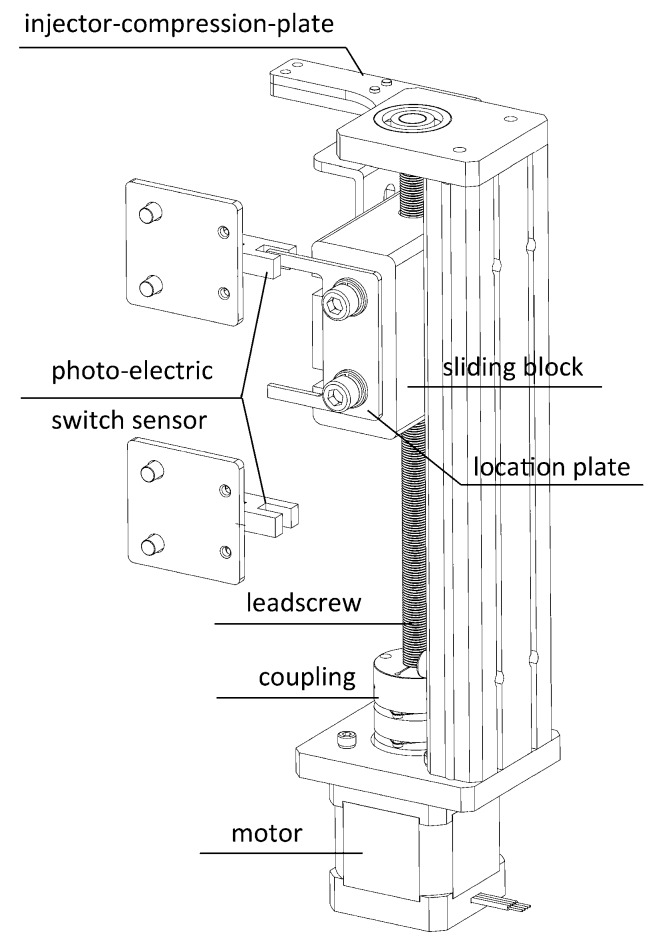
3D sketch of the automatic sample injection module.

Figure 6 shows a 3D sketch of the automatic sample injection module.

The main components of this module included a motor under the base of the instrument, a coupling connected the shaft of the motor and a leadscrew together, with a sliding block on the thread connected with the leadscrew which could lift up and down upon the spinning of the leadscrew. 

On the sliding block, two function plates were fixed with hex bolts. One was the injector compression plate right above the injectors. This plate could rise and fall in sync with the action of the sliding block to press the plunger of the injector down to push the sample out into the tube. The other one was the location plate of sliding block, functioning with two photo-electric switch sensors installed in the front surface of the back of the shell. This location plate is “U”-shaped, and two “hands” could trigger these two switches. In the up and down motion, the moment the upper photo-electric switch sensor was triggered by the upper “hand”, the motor would be stopped instantly by the microcontroller. This happened similarly when the lower photo-electric switch sensor was triggered by the lower “hand”. Thus, the rising and falling motion of this sliding stage could be restricted in this interval. In such an interval, the injector would be pressed just to the bottom, without overloading, which may damage the injector or be insufficient to leave residue.

##### Optical Fiber Probe Controller Module

**Figure 7:**
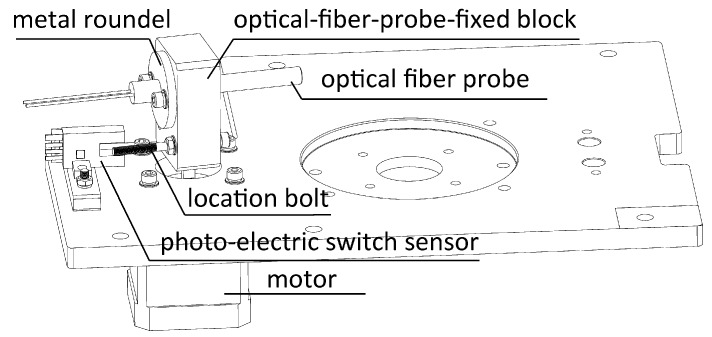
3D sketch of optical fiber probe controller module.

Figure 7 shows a 3D sketch of the optical fiber probe controller modules.

The optical fiber probe was fixed to the optical fiber probe fixed block by hex bolts through the screw holes on the metal roundel of this customized probe. The optical fiber probe fixed block was fixed on the shaft of the motor so that the optical fiber probe could be driven to revolve in sync with the motor. A location bolt was fixed on this block slightly below the probe used for orientation. During the spinning of this module it was able to trigger the photo-electric switch sensor installed nearby and, at that time, this module would be stopped. In that case, the optical fiber probe would precisely point at the tube under detection.

### 3.2. LabVIEW-Based Software

Software plays a critical role in this system. Firstly, the operator could command the action of the mechanical unit and optical-measurement unit through the software, and, additionally, this software was capable of managing the acquired spectral data, returning the final detection result.

#### 3.2.1. Programming Language Selection

LabVIEW was selected as our programming language. Compared with other languages, such as C++ or Visual Basic, a major advantage of LabVIEW is its rich graphic user interface (GUI) widgets and hardware drivers [30]. In this system, Ocean Optics offers a number of VIs relevant to spectra acquisition, together with a strong support for the communication between hardware and software. Additionally, LabVIEW could provide a useful toolkit, named advanced signal processing, based on which engineers could accomplish the program of spectral data processing by just using the ready-made functions. In short, its graphic nature makes it ideal for measurement and automation [31]. This software was developed in the environment of LabVIEW 2012 offered by NI.

#### 3.2.2. Design Criteria

To satisfy the requirements and to facilitate the development, this software was written in a modular manner, mainly including two core modules. The first was for the control of the mechanical unit and the other was used to cope with the spectral data.

**Figure 8:**
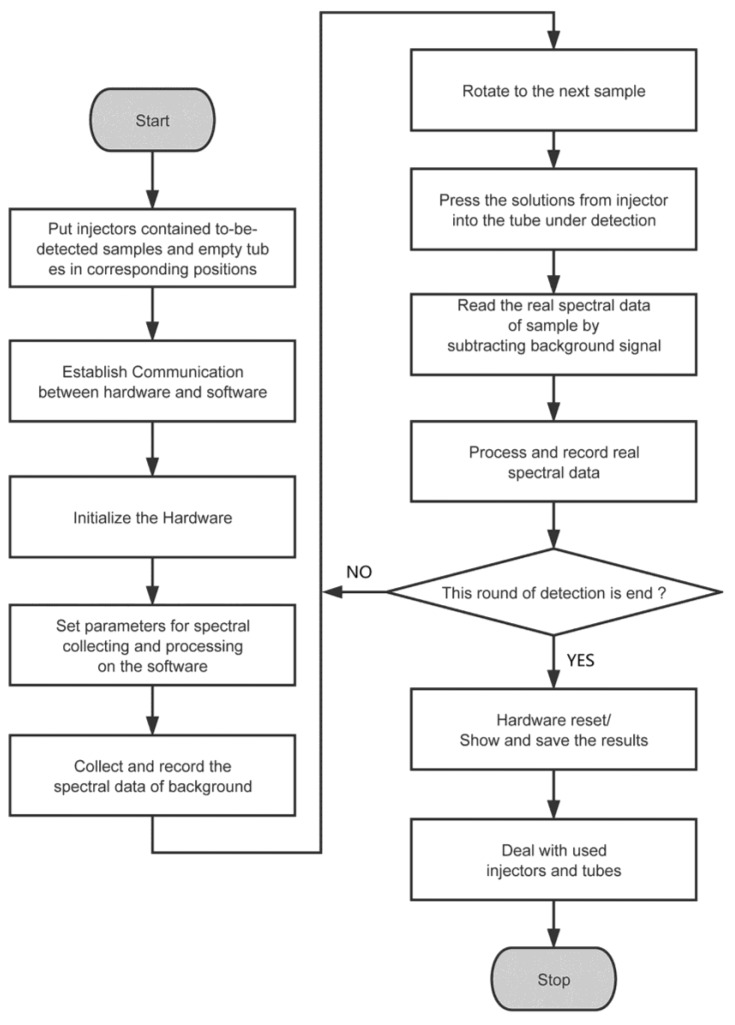
The typical flowchart of this software.

A typical flow chart of this software is illustrated in Figure 8. To start a detection, the operator would put in several single-use injectors that have already imbibed moderate samples and the same number of empty tubes, then set appropriate parameters and initialize the whole instrument system. After that, the system would work to obtain the spectral signals and then manage the result from them. At the end, when all of the samples in this run had been detected, the system would revert to its initial status. Detection results would be shown in the panel and saved on the computer automatically.

#### 3.2.3. Work Mode Selection

For simpler use in practice, there are two operation modes designed for the operator to select in light of specific purpose, manual mode and automatic mode. 

In manual mode, the operator could control the system to work step by step, redoing or skipping any step if necessary. It could be more flexible and controllable since every move would not start until the click on the specific button by the operator, suitable for some unusual tasks different from the standard detection process. For example, in testing a new sample to attempt appropriate parameters for spectral processing, this manual mode would be more suited. 

With regard to the automatic mode, the whole procedure of detection could be done automatically, as illustrated in Figure 8. The final result would also be saved on the computer by the software itself. This mode was much more suited for a repetitive and high-throughput detection for a certain kind of sample.

## 4. Results and Discussion

### 4.1. System Installation

**Figure 9:**
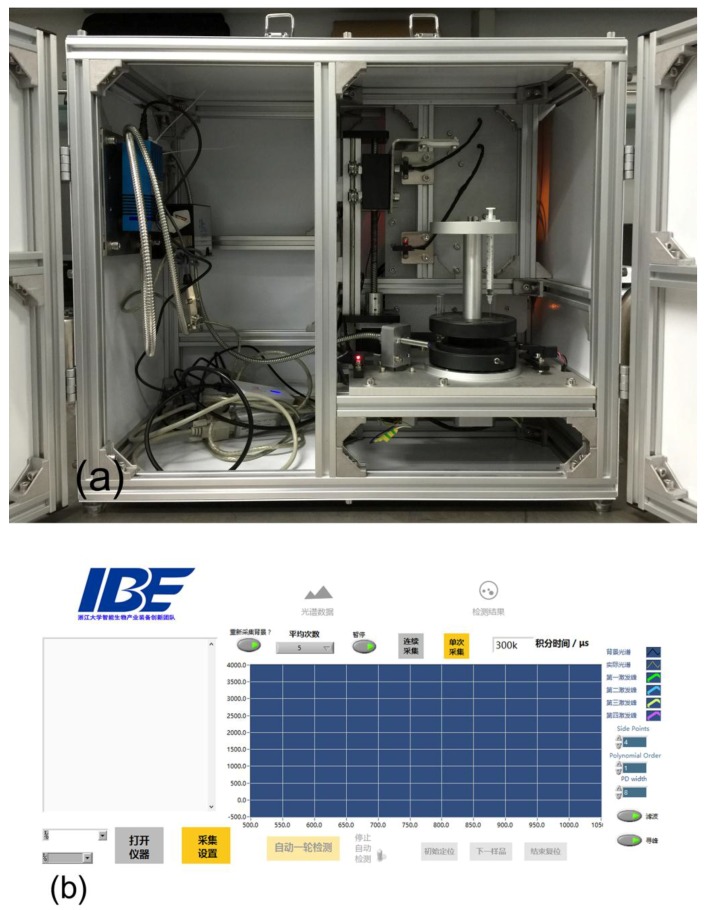
(**a**) Overall arrangement of all of the components inside the shell. (**b**) The main user interface of this software program, where most assignments including spectrum collection, display, and system control are done.

Figure 9a presents the overall arrangement of all the components inside. The light source and the fiber optic spectrometer were fixed on the left wall of the instrument, connected together by the optical fiber probe. The whole mechanical unit was placed in the right side of the instrument. The power supply cables and USB communication lines went out from the instrument through a small hole on the bottom-rear of the unit. The hardware was covered by an aluminum extrusion-made shell, blocking out light from the environment. Figure 9b shows the main user interface of the LabVIEW-based software and this software had been installed in the notebook computer in advance.

### 4.2. Spectrum Acquisition Parameters

In the original spectral signal collecting, ‘integration time’ and ‘average times’ are two critical parameters that should be adjusted by hand in advance. ‘Integration time’ is the length of the total time for the fiber-optic spectrometer to continuously collect the optic signal. A higher ‘integration time’ means a higher sensitivity to weaker optic signals, but it also might obtain some interference light. ‘Average times’ means the times of the original spectral signals that should be collected before averaging. Referring to the experience from the previous research [20] in which the core component of the optic signal collecting and the sample to be detected are quite similar, 300 ms was used as the integration time and 3 was used as the average times. 

**Figure 10:**
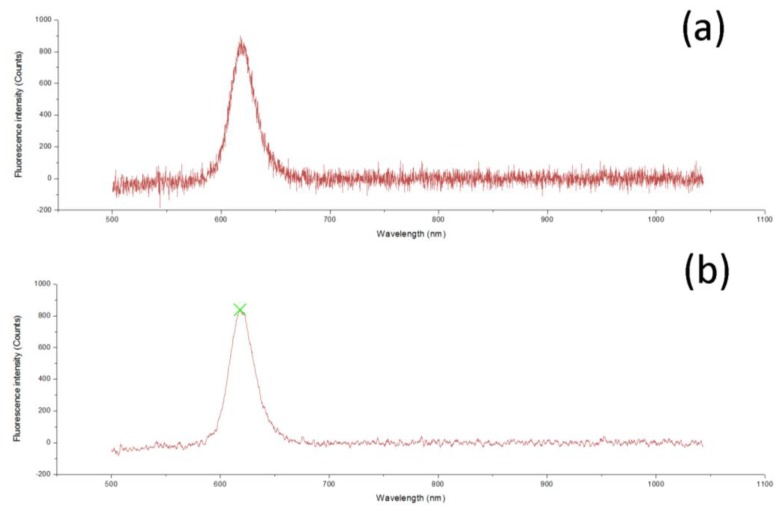
(**a**) Before and (**b**) after the use of the Savitzky-Golay filter and WA Multiscale Peak Detection. A green cross is used to mark the peak found by this algorithm.

Other than the random error that could be reduced by proper average times, there was another type of error in the spectrum curve, as shown in Figure 10a, making the signal too coarse to read. A Savitzky-Golay filter was selected as the smoothing algorithm. In addition, a wavelet-based method for automatic peak-finding offered by NI, named WA Multiscale Peak Detection, was also added to search for the peak. 

A comparison of the spectrum curve before and after processing are presented, respectively, in Figure 10a,b, revealing a readable curve and the peak with a cross mark.

### 4.3. System Test

For the elementary evaluation of this instrument system, pure QD solutions were used as samples instead of bacterial pathogens. As QD is the only component that could emit strong and stable fluorescence in the bacteria detection, using pure QD solutions could simulate actual conditions and circumstances in detection. Additionally, pure QD solutions could be prepared more uniformly by simple manual operation, reducing the error in system testing caused by samples.

#### 4.3.1. Blank Test

To verify the operation of this instrument system, we performed a test using a blank reference to obtain quantitative information regarding the reproducibility in one detection station and between all ten. 

**Figure 11:**
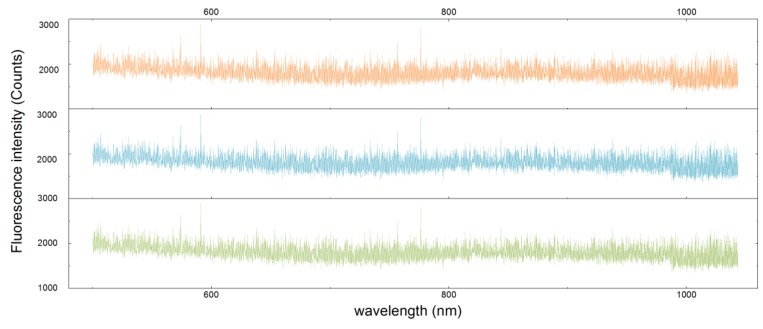
Three spectra curves randomly selected from the test.

Ten empty tubes and ten injectors with 0.1 mL of PBS were placed into the rotation stage, and the parameters related to data processing were settled as described in Section 4.2. To eliminate the interference of the light source whose center wavelength is near 460 nm, the range of the spectrum curve was limited to between 500 nm to 1040 nm. Three spectra curves selected randomly from the test are presented in Figure 11. The tendency and amplitude of them seemed to be quite approximate.

**Figure 12:**
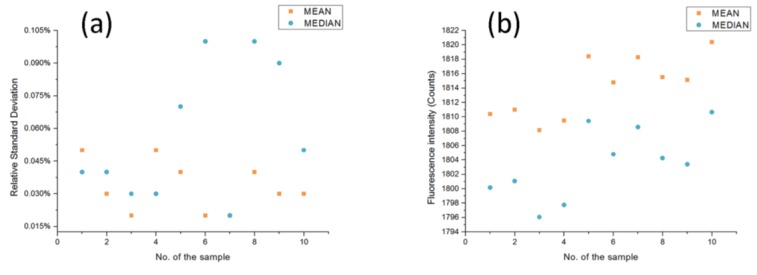
(**a**) Comparison of the R.S.D. of the mean and median for the spectra data in a specific detection station. (**b**) Comparison of means and medians for each list of spectra data from ten detection stations.

For a quantitative comparison, means and medians of all of these lists of spectra data were calculated. As for each sample, measurements were repeated six times. The comparison of relative standard deviation (RSD) of these means and medians for each curve is represented in Figure 12a. Afterwards, the comparison in the form of fluorescence intensity of the means and the medians between these ten samples in ten different detection stations was also done and the result is shown in Figure 12b.

As can be seen from the figure, the mean and median of the data from the specific detection stations were quite impressive, with a few fluctuations; the RSD, which ranged from 0.02% to 0.11%, suggesting this home-made system had outstanding stability for the detection in a specific sample. The error might be caused by the variation of environmental temperature and the systematic error of the fiber optic spectrometer.

The reproducibility between these ten different tubes was also inspiring. As depicted in the figure, the mean and median for every list of spectrum data in the form of fluorescence intensity were quite close, and the RSDs of the average and median were 0.23% and 0.27%, respectively, by calculation, slightly higher than that of the previous test. It could be attributed to the fact that there are more factors causing error in this case, as in the detection for a specific sample, where the influence made by the tube would not be taken into consideration. That is, things such as a slight deviation of the distance between the optical fiber probe and the tube, the difference of every tube, and different surface finishes or thicknesses could contribute to accumulating more deviations in the detection. However in short, it can be concluded from these two tests that the error between different tubes was quite small, which means that these ten detection stations could be treated as the same one in real detection. To conclude, this instrument system has a high reproducibility both in a specific detection station and between different detection stations.

#### 4.3.2. QD Test

##### Measurement Conditions

To evaluate this instrument system in practical detection, several tests were performed, including single type QD solutions and a mix of three types of QD solutions. The QDs used in these tests were prepared separately from different batch products, but had the same emission wavelengths of 528 nm, 572 nm, and 621 nm, respectively. Four tubes of samples for repeating were used in each run of detection and the detection stations used in this home-made instrument were fixed between the first and the forth.

In the single type of QD solution test, different concentrations from 2 × 10^−3^ μM to 1 × 10^−2^ μM (diluted from 500 to 100 times) of three kinds of pure QD solutions were used, while in the mixed test, the concentrations for each kind of QD sample was from 6.67 × 10^−4^ μM to 3.33 × 10^−3^ μM (diluted from 1500 to 300 times). As a reference, all of the samples of the same quantity and concentrations were also detected in parallel by the Synergy™ H1 for a contrast.

##### Comparison Study Result

**Figure 13:**
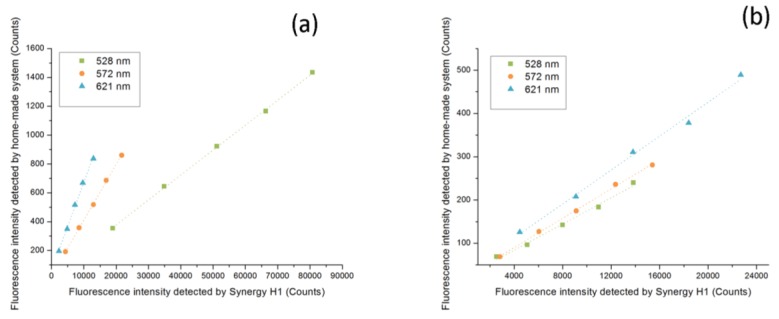
(**a**) Correlation tests for each single-type QD solution between these two instruments at the wavelength of 528 nm, 572 nm, and 621 nm, respectively; (**b**) Correlation tests for the mixed samples.

Figure 13a,b show the correlation of the detection results in terms of the intensity of optic signal between this home-made instrument system and the Synergy™ H1. The correlation coefficient (R value) was 0.99974, 0.99973, and 0.99847 for each type of single QD solutions, and the results for the mixed samples were 0.99596, 0.99939, and 0.99723 in three wavelengths, respectively, all of which were at a high level, suggesting a similar performance between these two instruments at these three wavelengths, though in different orders of magnitude in the recording of fluorescent intensity.

**Figure 14:**
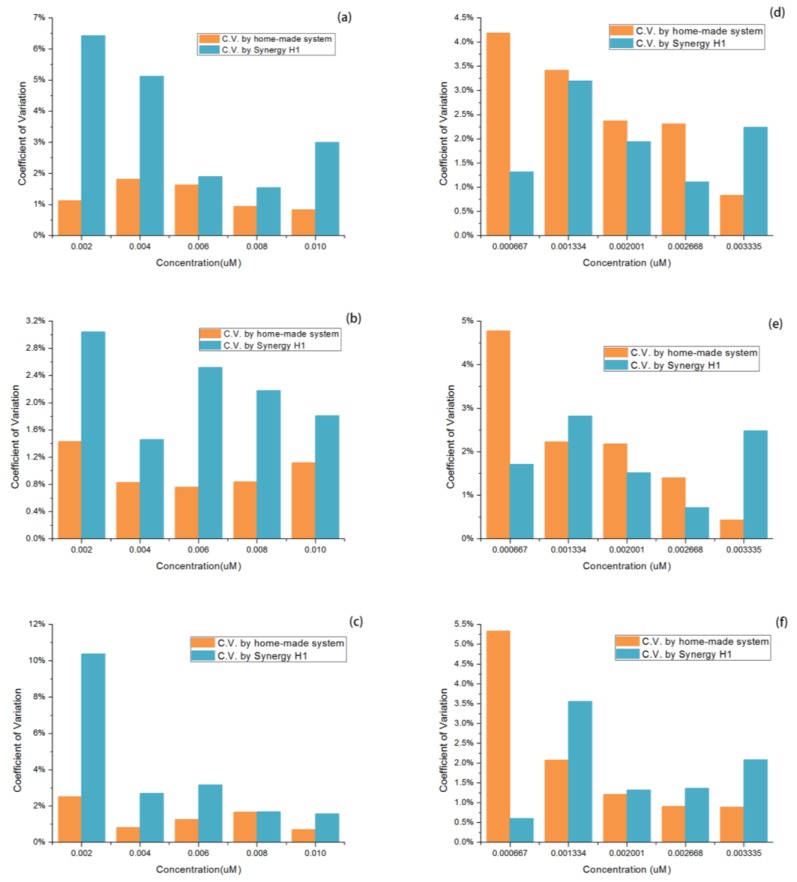
(**a**–**c**) Comparison of C.V. between these two systems with a single-type QD solutions test. (**d**–**f**) Comparison of C.V. between these two systems in the mixed-QD solutions test.

More linear calibrations were obtained, as shown in Table 1 and Table 2. The contrast about the reproducibility for the same concentrations between the four repeats based on the value of coefficient of variation for all are shown in Figure 14a–f.

As can be seen, the linear correlation of this home-made instrument and the Synergy™ H1 was close, both at a high level. More exactly, this home-made instrument performed better in the test by the single QD solutions while the Synergy™ H1 was more impressive in the test of mixed QD samples. This could be attributed to the higher accuracy for the Synergy™ H1 at rather low concentrations. Yet with this slight difference, both apparatus behaved outstandingly in linear correlation tests. 

The sensitivity of the Synergy™ H1 seemed to be higher than that of this home-made instrument, as shown by the higher calibration slopes. However, because of the difference in the recording of optical signals between these two systems, it might not be appropriate to directly compare them quantitatively.

In the contrast of the reproducibility of these four repeats in each concentration, the result was slightly similar with the circumstances of those in linear calibrations. In a lower concentration of QD solutions, the C.V. of nearly all three kinds in all of the concentrations acquired by this home-made instrument was lower than that from the Synergy™ H1, suggesting an overwhelmingly higher reproducibility of this home-made instrument. As a matter of fact, in actual in-situ detection, this range of concentration of QD solutions is most frequently used. When the concentration of QD samples lowered to the range of 6.67 × 10^−4^ μM to 3.33 × 10^−3^ μM (diluted from 1500 to 300 times), the Synergy™ H1 showed a more stable performance in reproducibility, even better in some concentrations. However, this home-made instrument performed with more fluctuation with the decline of concentration, and the distinction between these two was the greatest at the lowest concentration. This can also be explained in that the Synergy™ H1 has a better accuracy at rather low concentration.

#### 4.3.3. Typical Food-Borne Bacteria Test

**Figure 15:**
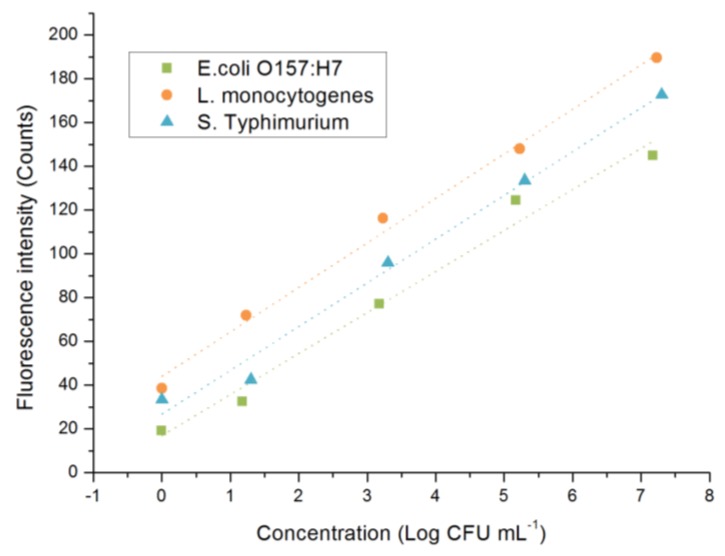
The linear relationship of *E. coli* O157:H7, *L. monocytogenes*, and *S. Typhimurium* at the concentration range from 10^0^ to 10^7^ CFU·mL^−1^ with the fluorescence intensity measured by this home-made instrument. The equations are y=18.76x+16.93, $R^{2}=0.98$ (*E. coli* O157:H7), y=20.36x+43.95, $R^{2}=0.99$ (*L. monocytogenes*), and y=20.00x+26.76, $R^{2}=0.99$ (*S. Typhimurium*), respectively.

As introduced in Section 2.2, three typical kinds of food-borne bacteria, *E. coli* O157:H7, *L. monocytogenes*, and *S. Typhimurium*, were chosen to prove that this instrument system is able to detect bacteria. The test samples containing three kinds of bacteria together had been prepared from 10^0^–10^7^ CFU·mL^−1^. Three repeats for each sample were done and the linear relationships between the cell population (converted to log values) and the fluorescence intensity for each pathogen are presented in Figure 15.

As shown in Figure 15, with the increase of the concentration of the bacteria, the fluorescence intensity measured by this home-made instrument system would also increase. The linear relationship between them were all at high levels for each kind of detected pathogen. Together with other linear calibrations, such as intercept and slope, all are close to our previous experiment results [20]. The limit of detection (LOD) was calculated by multiplying the standard deviation of the result of the negative control by three, and based on this algorithm, the LODs of these three types of food-borne pathogens could be calculated to be about 350, 98, and 267 CFU·mL^−1^, which are also similar to our previous results [20]. It could be inferred that the design and the construction of this home-made instrument system incurs no negative interference to the detection. In other words, this system is sensitive and stable enough for bacteria detection, at least in the laboratory circumstance [32].

### 4.4. Discussion

To sum up, the detection result of this home-made instrument system was credible according to the high correlation coefficient (r) with the result by the Synergy™ H1. Furthermore, this system offered slightly improved, and quite better, performance in linear correlation and repeatability in the concentration range of QD solutions from 2 × 10^−3^ μM to 1 × 10^−2^ μM (diluted from 500 to 100 times), the range of which is the most frequently used in real bacteria detection. Yet when it came to a lower concentration 6.67 × 10^−4^ μM to 3.33 × 10^−3^ μM (diluted from 1500 to 300 times), the commercial product was able to do better than the home-made one. However, the distinction between them was still quite small. It could be concluded that in the measurement of pure fluorescence signals without pathogens, this home-made system performs as outstanding as the lab instrument. In bacteria tests, this home-made system was able to perform closely to our previous laboratory experiments. This could prove that this newly designed instrument is sensitive and stable enough for pathogen detection in food samples. As for the in-situ use, some preliminary tests of food-borne pathogens detection in food matrix had been done and the results were described in detail in another manuscript by our group [32]. These results showed the outstanding capability of this home-made system compared with gold standard culture plating method.

In the point of instrumental study, there are other more advantages of this home-made system compared with the commercial product Synergy™ H1 for in-field use other than performance. Firstly, this home-made system could finish fluorescence signal collection, data analysis including smoothing and peak detection, data recording, and results calculation in nearly 20 s per sample (integration time was 300 ms, and average times was 3) with 0.2 nm interval of wavelength in spectrum. It took the commercial one the same time to do so with 5 nm wavelength interval and 30 to 60 s with 1 nm interval to finish detection for each sample. It could be attributed to the use of the spectrometer USB4000 that is able to acquire complete spectrum from 200 to 1100 nm in only one time of optical signal collection. While for the Synergy™ H1, it could only acquire spectrum signal at a specific wavelength because of the use of excitation filters thus it would take much more times if the range of interested wavelength is wide. Although this commercial one could detect 384 samples once while this home-made system could support only 10 to the most at a time. Considering the faster rate of detection of our instrument and the fact that 10 samples for one run are enough in most cases for on-site detection based on our experience, we do not think it is a disadvantage especially that portability is an important factor. In addition, the cost of this instrument system is much lower than this commercial one: 6000 US dollars V.S. 48,000 US dollars. The cost for the detection for each sample is nearly equal for these two instruments that is estimated to be nearly 10 US dollars [32], considering the dosages for QD, bacteria sample, and other reagents are similar. Yet, we have to admit that this home-made system has similar volume and weight with this commercial one: 50.2 × 42.4 × 27.5 cm V.S. 39.1 × 42.2 × 32.8 cm and 15 kg V.S. 22.5 kg, which are not portable enough for use on the spot. The improvement of miniaturization would need to be done in our next work. In the fact, we had considered and left room for improvement in this first version. The room for placing spectrometer and light source could be narrowed, and the optical fiber probe could be shorten. The electrical source 220 V AC could be replaced by a 12~24 V battery which is supported by the motors and circuits we used. The power of motors could be reduced for a lighter weight. We believe these would work as there were a lot redundancies left to ensure the performance in the design of the first version. And these are needed to be promoted in our future work.

## 5. Conclusions

We have presented a novel, highly-automated instrument prototype for rapid and high-throughput detection of pathogens on the spot based on fluorescence detection. This system was constructed by commercial off-the-shelf optical components for the collection of optic signals, and a self-designed mechanical unit served as the power supply to drive the hardware. Additionally, a software based on LabVIEW was developed as an interface for the operator to command the whole system and, meanwhile, this software could complete data recording, analysis, and presentation. With the co-operation of these units, this instrument system is able to automatically measure ten samples at most in each run of detection.

For validation, pure QD solutions and bacteria samples were used in the test. Finally, this home-made system could offer a comparative performance with the Synergy™ H1 commercial product with a much lower cost and a faster detection rate. Furthermore, we have proven that this newly-designed instrument system resulted in no more negative influence in bacteria detection and it is capable for pathogen detection in low concentrations in food samples compared with our previous experimental results [20]. Though some upgrades and further harder evaluations need to be done to make this system more portable and suited for on-site use. With advantages of low-cost, easy-to-use, outstanding performance and high-throughput, we think it great potential to be used in practice in the on-site detection for food-borne pathogens.

## Figures and Tables

**Table 1:** Comparison of linear calibrations between these two systems in single-type QD solution tests.

Parameter ^1^	Home-Made			Commercial		
Wavelength (nm)	528	572	621	528	572	621
Intercept	99.14	22.87	32.75	3937.8	–54.34	–451.18
Slope	1.34 × 10^5^	8.33 × 10^5^	8.01 × 10^5^	8.00 × 10^6^	2.00 × 10^6^	1.00 × 10^6^
R^2^	0.9989	0.9998	0.9997	0.9993	0.9992	0.9962

^1^ the linear range in this test of each QD sample is from 2 × 10^−3^ μM to 1 × 10^−2^ μM (diluted from 500 to 100 times).

**Table 2:** Comparison of linear calibrations between these two systems in testing mixed QD solutions.

Parameter ^1^	Home-made			Commercial		
Wavelength (nm)	528	572	621	528	572	621
Intercept	33.29	17.52	17.34	–43.93	–289.61	–456.13
Slope	4.49 × 10^5^	2.66 × 10^5^	2.15 × 10^5^	2.00 × 10^6^	2.00 × 10^6^	1.00 × 10^6^
R^2^	0.9956	0.9981	0.988	0.9997	0.9999	0.9993

^1^ the linear range in this test of each QD sample is from 6.67 × 10^−4^ μM to 3.33 × 10^−3^ μM (diluted from 1500 to 300 times).
